# Association Between Post-Trigger Hormones and *In Vitro* Fertilization Cycle Outcomes

**DOI:** 10.14740/jcgo1582

**Published:** 2026-02-08

**Authors:** Olivia B. Chafitz, Anat Chemerinski, Swata Gade, Evelyn Minis, Patricia Greenberg, Peter G. McGovern, Sara S. Morelli

**Affiliations:** a Department of Obstetrics, Gynecology and Reproductive Health, Rutgers New Jersey Medical School, Newark, NJ 07103, USA; b Department of Biostatistics and Epidemiology, Rutgers School of Public Health, Rutgers University, Piscataway, NJ 08854, USA

**Keywords:** Infertility, Ovarian stimulation, Assisted reproductive techniques, Estradiol, Progesterone

## Abstract

**Background:**

Numerous studies have evaluated the association between estradiol and progesterone levels on the day of trigger administration and cycle outcomes; however, less is known about serum hormone levels following trigger and whether they are associated with cycle outcomes. This study was conducted to assess the association between serum steroid hormone parameters on the day after oocyte maturation trigger and ovarian stimulation cycle outcomes.

**Methods:**

This was a retrospective cohort study. All patients undergoing ovarian stimulation with the use of gonadotropins between September 2018 and September 2020 were assessed for inclusion. Chart review was performed to collect demographic information, cycle and trigger type, pre- and post-treatment serum hormone levels (estradiol, progesterone), and cycle outcomes. The primary outcomes were oocyte recovery rate, oocyte maturity rate, oocyte fertilization rate, and blastulation rate.

**Results:**

A total of 320 cycles were included in the study from 245 unique patients; 19% of cycles utilized a gonadotropin-releasing hormone (GnRH) agonist stimulation protocol and 81% utilized a GnRH antagonist stimulation protocol. Post-trigger progesterone level was associated with poor oocyte recovery, but not with oocyte maturity, fertilization, or blastulation rates. Post-trigger estradiol levels were not associated with oocyte recovery rate, oocyte maturity rate, fertilization rate, or blastulation rate.

**Conclusions:**

This is one of few studies evaluating the association between estradiol and progesterone levels on the day after trigger injection and oocyte stimulation cycle outcomes. Our findings suggest that the association between post-trigger progesterone levels and poor oocyte recovery rate may be small but meaningful.

## Introduction

The pursuit of *in vitro* fertilization (IVF) is a time-consuming, emotionally taxing, and expensive process for patients. Predicting IVF success is therefore of great value to both clinicians and patients. Two commonly used models—the Society for Assisted Reproductive Technology (SART) Patient Predictor and the Univfy PreIVF Report—can be used to predict pregnancy based on pre-cycle variables such as age, height, weight, reproductive history, clinical diagnosis, and ovarian reserve testing results [1, 2]. Recent studies have evaluated predictive models that also utilize in-cycle variables, such as peak estradiol, number of oocytes recovered, and number of embryos cryopreserved, allowing for real-time adjustments to predicted IVF cycle success rates [3]. Identification of incycle variables associated with poor cycle outcomes can help clinicians as they counsel patients and attempt to manage expectations for IVF cycles. Furthermore, if associations exist between post-trigger hormone levels and cycle outcomes, clinicians might be able to use post-trigger labs to guide decisions around cycle cancellation or to modify future treatment cycles.

Routine monitoring during ovarian stimulation (OS) involves serial ultrasounds and serum hormones, including serum concentrations of estradiol (E2) and progesterone (P4). This pre-trigger monitoring is crucial to ensure appropriate follicular development and optimize timing of oocyte retrieval. Pre-trigger estradiol is assessed to determine a patient’s response to gonadotropins, as well as their risk of developing ovarian hyperstimulation syndrome (OHSS), a severe and iatrogenic complication of OS. Accordingly, estradiol levels on the day of trigger administration vary significantly, largely based upon the number of mature oocytes present. Studies have confirmed that the estradiol level rises in direct relation to the total number of oocytes [4, 5] and mature oocytes [4]. Progesterone levels are often monitored throughout OS cycles as well. When levels rise before the oocyte maturation trigger has been administered, fresh embryo transfer outcomes may be impacted, likely due to the prolonged exposure of the endometrium to progesterone. Poor cycle outcomes [6] including decreased clinical pregnancy rates [7–9] and live birth rates [8] have been described in association with elevated pre-trigger progesterone levels.

In contrast, there is a paucity of data surrounding the utility of post-trigger hormones. Serum human chorionic gonadotropin (hCG) and luteinizing hormone (LH) levels may be measured on the day after injection of hCG and gonadotropin-releasing hormone (GnRH) agonist triggers respectively, to ensure proper administration; failure to administer the trigger injection appropriately can result in a poor oocyte yield or oocyte immaturity at the time of retrieval. Though estradiol and progesterone are not often assessed on the day after trigger injection, they may provide valuable insight regarding the patient’s response to the protocol and aid clinicians as they plan for subsequent cycles. There are limited, and somewhat contradictory, data regarding the association between post-trigger estradiol levels and cycle outcomes. While some studies have found a positive association between post-trigger estradiol and IVF cycle outcomes [10], others have found a negative association [11, 12], and still others report no effect [12–14]. Even fewer studies have examined the predictive value of post-trigger progesterone in freeze-all cycles [15, 16], and no clear consensus exists. Understanding the association between hormone levels on the day after oocyte trigger and cycle outcomes can aid clinicians in managing expectations for the cycle.

To address a critical gap in the literature regarding the clinical utility of post-trigger hormone assessment, this study evaluated whether serum estradiol and progesterone levels measured on the day following oocyte maturation trigger are associated with OS cycle outcomes. We specifically hypothesized that lower post-trigger estradiol and progesterone levels would be associated with poor IVF cycle outcomes including reduced oocyte recovery, maturity, fertilization, and blastulation rates. By clarifying the associative value of post-trigger hormone levels, this study aims to inform clinical decisionmaking regarding the early identification of suboptimal cycles and the potential role of post-trigger monitoring in optimizing IVF cycle management.

## Materials and Methods

### Study population

This was a retrospective cohort study at an academic-affiliated private practice. This study was approved by the Rutgers University Institutional Review Board (Study ID: Pro2021000867), and the need for informed consent was waived because of the retrospective nature of the study. All patients aged 18 or older undergoing OS cycles with the use of gonadotropins for IVF from September 2018 through September 2020 were assessed for inclusion. Patients undergoing cycles using agents other than gonadotropins, donor oocyte cycles, and oocyte cryopreservation cycles were excluded. Chart review was performed to collect demographic information, cycle and trigger type, pre- and post-treatment serum hormone levels (estradiol, progesterone), and cycle outcomes. This study was conducted in compliance with the ethical standards of the responsible institution on human subjects as well as with the Helsinki Declaration.

### OS protocol

Follicular growth was monitored by ultrasound and serum hormone measurements. Follicular growth was considered sufficient when ultrasound monitoring revealed at least two follicles with a mean diameter of 18 mm or larger. Patients were instructed to administer an intramuscular injection of hCG trigger (10,000 IU of hCG), leuprolide acetate trigger (two doses of leuprolide acetate 80 units every 12 h plus 1,000 IU of hCG) or a dual trigger (10,000 IU of hCG plus two doses of leuprolide acetate 80 units every 12 h) for final oocyte maturation. Oocyte retrieval was scheduled for 34 hours after the first injection. All patients were instructed to return for a blood draw between 7:00 and 8:00 am in the morning following the injection, to confirm that the trigger medication had been properly administered (6 ± 2 h later). Serum hCG, E2, P4, folliclestimulating hormone (FSH) and LH were assessed by chemiluminescent assay (Immulite 1000, Siemens, Deerfield, IL).

### Assessment of IVF cycle outcomes

Recovery rate was defined as the number of oocytes retrieved divided by the number of follicles > 12 mm seen on ultrasound on the day of trigger. Given the strict practice of the clinic providers to count, measure and document all follicles > 12 mm, this was considered a reliable measure. Maturity rate was defined as the number of metaphase II (MII) oocytes over the number of total oocytes retrieved. Fertilization rate was defined as the number of embryos with two pronuclei (2PN) over the total number of MII oocytes, and blastulation rate was defined as the number of blastocysts over the number of 2PN embryos. Pregnancy rate was not assessed due to the high percentage of cycles in which all embryos were frozen and/or preimplantation genetic testing (PGT) was performed. A priori, poor IVF results were defined as: an oocyte recovery rate ≤ 50%, a maturity rate ≤ 40%, a fertilization rate ≤ 75%, and a blastulation rate ≤ 20% when four or more oocytes were fertilized, based on studies [17–20] in which similar or higher cutoffs were reported. Where possible, lower thresholds were intentionally used, as cycle cancellation is typically reserved for situations in which markedly poor outcomes are anticipated.

### Statistical methods

All study measures, including patient demographics, were first summarized using mean and standard deviation (SD), median with interquartile range (IQR), or as a frequency and percentage, as appropriate. This was done for the entire patient sample, as well as by the primary stimulation type (agonist vs antagonist). In order to determine the effects of post-trigger estradiol and progesterone levels on the occurrence of poor IVF outcomes, a series of separate multivariable generalized estimating equations (GEE) models with exchangeable correlation structures (additionally adjusted for patient age at cycle start, body mass index (BMI), race/ethnicity, trigger type, and stimulation type, where indicated) were fit, where a two-sided P value < 0.05 was considered statistically significant. All statistical analyses were performed using R Version 4.2.2 software.

## Results

### Demographic characteristics and cycle outcomes

A total of 245 patients who underwent a total of 320 cycles met the inclusion criteria for this study. Seventy-five percent of patients are represented by one cycle, 20% are represented by two cycles, and the remaining 5% are represented by up to 4 cycles. Patients’ ages ranged from 25 to 48 years with a mean (SD) age at the start of the cycle of 37.6 (4.1) years and the mean (SD) BMI was 27.7 (8.1). The mean (SD) number of oocytes retrieved was 11.0 (7.5), and the oocyte recovery rate mean (SD) was 90% (30%). The mean (SD) number of mature oocytes per cycle was 7.2 (5.1) with an average maturity rate of 70% (20%), and the mean (SD) number of oocytes fertilized per cycle was 6.2 (4.4) with an average fertilization rate of 90% (10%). In 82.5% of cycles, embryos progressed to the blastocyst stage, with a mean (SD) of 2.9 (3.0) blastocysts per cycle and a mean (SD) blastulation rate of 50% (30%). Demographic characteristics and cycle outcomes are shown in Tables 1 and 2, respectively.

### Post-trigger progesterone level is associated with low oocyte recovery rate

**Figure 1.**
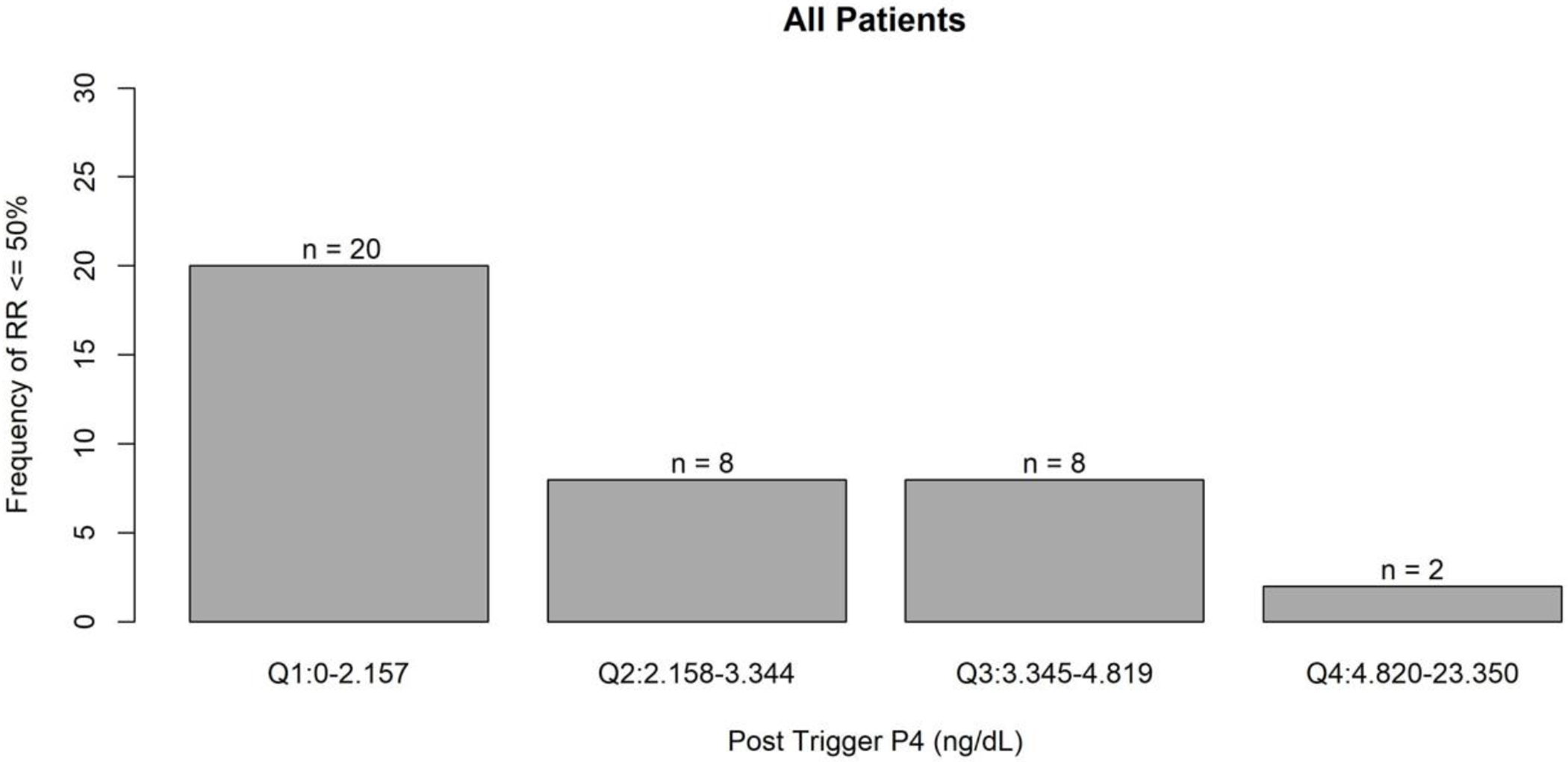
Frequency of recovery rate ≤ 50% associated with post-trigger progesterone levels. Q1: quartile 1, Q2: quartile 2, Q3: quartile 3, Q4: quartile 4, P4: progesterone.

**Figure 2.**
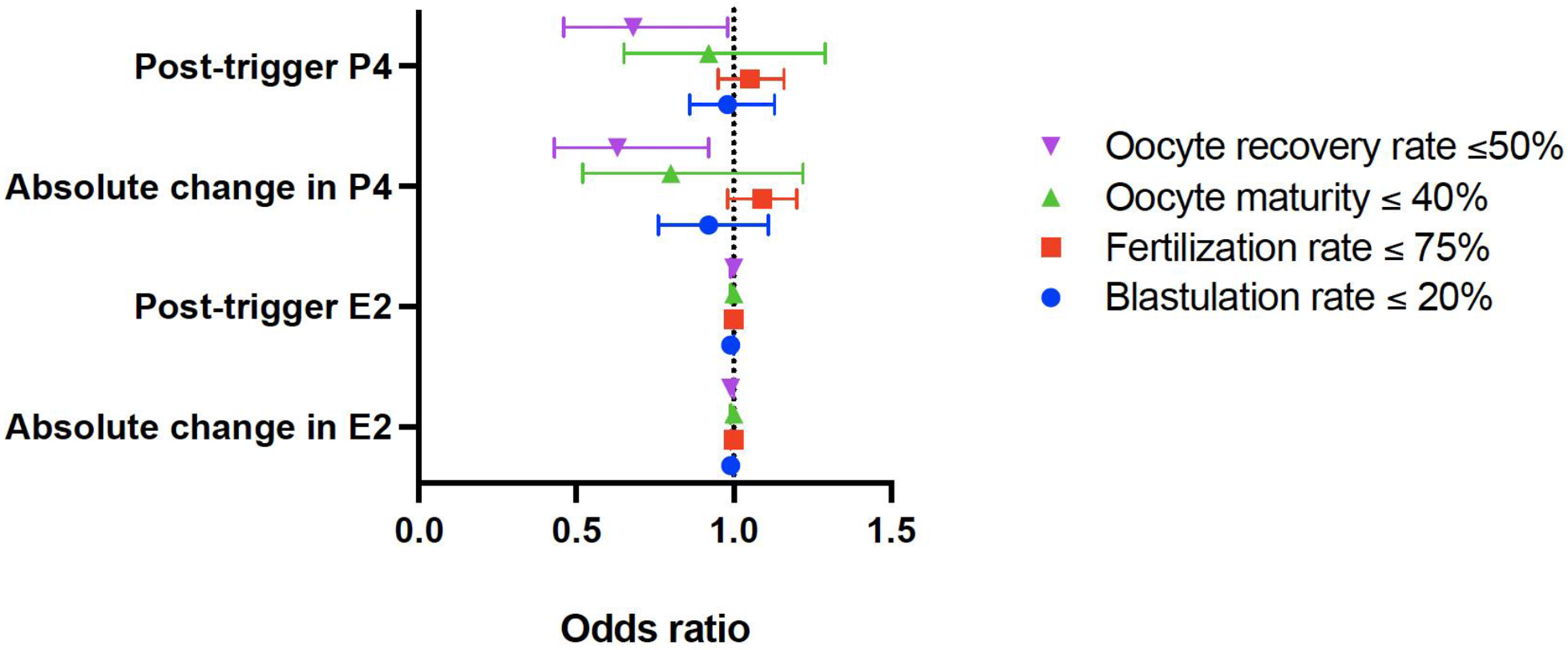
Forest plot depicting the odds ratio for IVF outcomes by post-trigger hormone parameters. IVF: *in vitro* fertilization; E2: estradiol; P4: progesterone.

Post-trigger changes in progesterone were then evaluated to determine if they were associated with poor IVF cycle outcomes. Results from a multivariable GEE model adjusted for age, BMI, race/ethnicity, post-trigger E2, trigger type, and stimulation type showed that each 1 ng/mL increase in post-trigger progesterone was associated with a 32% reduction in the odds of a low oocyte recovery rate ≤ 50% (odds ratio (OR) (95% confidence interval (CI)), 0.68 (0.46, 0.98), P = 0.04) (Fig. 1). Additionally, each 1 ng/mL increase in absolute post-trigger progesterone level was associated with a 37% reduction in the odds of a low oocyte recovery rate ≤ 50% (OR (95% CI), 0.63 (0.43, 0.92), P = 0.02) (Fig. 2).

### Post-trigger progesterone level is not associated with oocyte maturity rate, fertilization rate or blastulation rate

The impact of post-trigger progesterone on oocyte maturity and fertilization rates was assessed. In multivariable GEE models adjusted for age, BMI, race/ethnicity, post-trigger E2, trigger type, and stimulation type (maturity outcome) or for BMI, post-trigger E2, trigger type, and stimulation type (fertilization outcome), both measures used to analyze post-trigger progesterone (absolute level and absolute change) were not significantly associated with the outcomes of a maturity rate ≤ 40% (OR (95% CI), 0.92 (0.65, 1.29), P = 0.63; 0.80 (0.52, 1.22), P = 0.29) or a fertilization rate ≤ 75% (OR (95% CI), 1.05 (0.95, 1.16), P = 0.31; 1.09 (0.98, 1.20), P = 0.10). In cycles where four or more oocytes were fertilized, post-trigger progesterone was also not associated with a low blastulation rate (defined as a blastulation rate ≤ 20%) based on the results of the multivariable GEE models adjusted for age, BMI, race/ethnicity, post-trigger E2, trigger type, and stimulation type (OR (95% CI), 0.98 (0.86, 1.13), P = 0.80; 0.92 (0.76, 1.11), P = 0.37) (Fig. 2).

### Post-trigger estradiol level is not associated with poor IVF outcomes

Additionally, the impact of post-trigger estradiol levels on the four outcomes of interest (recovery, maturity, fertilization and blastulation rates) was assessed. In multivariable GEE models adjusted for age, BMI, race/ethnicity, post-trigger P4, trigger type, and stimulation type, both measures used to analyze post-trigger estradiol levels (absolute level and absolute change) were not associated with any clinically significant outcomes. Three of the four markers of poor cycle outcome were not statistically significant, including a recovery rate ≤ 50% (OR (95% CI), 1.00 (0.99, 1.00), P = 0.59; 0.99 (0.99, 1.00), P = 0.19), a maturity rate ≤ 40% (OR (95% CI), 1.00 (0.99, 1.00), P = 0.44; 1.00 (0.99, 1.00), P = 0.94), or a blastulation rate ≤20% when more than four oocytes were fertilized (OR (95% CI), 0.99 (0.99, 1.00), P = 0.08; 0.99 (0.99, 1.00), P = 0.09). While the odds of a fertilization rate ≤ 75% with increasing post-trigger estradiol was statistically significant (OR (95% CI), 1.0003 (1.00002, 1.0005), P = 0.03) (Fig. 2), the magnitude was extremely small and not clinically meaningful, and would not justify any change in clinical management or counseling.

Taken together, our results indicate that a lower post-trigger progesterone level is associated with poor oocyte recovery rate (retrieving oocytes from < 50% of follicles > 12 mm in mean diameter), and that post-trigger changes in estradiol are generally not associated with poor cycle outcomes.

## Discussion

Changes in hormone levels throughout OS cycles have been studied as possible predictors of cycle outcomes. Several studies have evaluated the association between pre-trigger endocrine profiles (obtained on the day of oocyte maturation trigger) and cycle outcomes such as pregnancy rates and live birth rates [8, 9]. There are fewer studies evaluating the association between post-trigger endocrine profiles (obtained on the day after oocyte maturation trigger) and cycle outcomes. We sought to determine whether progesterone and estradiol levels on the day after trigger injection were associated with cycle outcomes including oocyte recovery, maturity, fertilization, and blastulation rates. We found that low post-trigger progesterone level is associated with poor oocyte recovery rate and is not associated with any other measures of poor cycle outcome. Post-trigger estradiol level was not associated with any measures of poor cycle outcome.

A limited number of studies have assessed whether the change in estradiol between the day of hCG administration (pre-trigger) and the day after hCG administration (post-trigger) is predictive of cycle outcomes and none have identified a correlation [12–14]. Two retrospective analyses examined the change in E2 following oocyte maturation trigger administration and found no difference in cycle outcomes [12, 13]. In keeping with these findings, our study also did not identify a correlation between estradiol levels on the day of trigger and cycle outcomes, nor did we identify a correlation with estradiol on the day after trigger and any examined cycle outcomes.

Studies assessing the effect of elevated progesterone on the oocyte have arrived at contradictory conclusions. For example, a retrospective analysis performed by Woo et al assessed progesterone levels on the day of trigger administration and found that oocyte maturation rates were decreased when progesterone ≥ 2.25 ng/mL, fertilization rates were decreased when progesterone was ≥ 1.25 ng/mL, and the ratio of good quality embryos (GQE) at the cleavage stage as defined by the Gardner and Schoolcraft grading system was decreased when progesterone was ≥ 1.75 ng/mL [21]. Recent studies have also evaluated whether progesterone level is associated with top quality embryo (TQE) rate and found contradictory results. Embryos designated as TQE have certain characteristics that consistently correlate with improved cycle outcomes. Huang et al retrospectively analyzed 4,236 fresh IVF cycles and found that an elevated serum progesterone level (> 2.0 ng/mL) on the day of hCG trigger was associated with lower TQE rates [22]. Similarly, a retrospective analysis of 986 patients found that elevated levels of progesterone on the day of oocyte maturation induction was associated with a decreased top quality blastocyst formation rate [23]. Contrastingly, Baldini et al performed a retrospective analysis of 131 patients undergoing frozen embryo transfer (FET) and compared cycle outcomes among those with an elevated progesterone on the day of hCG administration (≥ 1.2 ng/mL) and patients without elevated progesterone (< 1.2 ng/mL) [24]. They found no significant difference in oocyte recovery rate, fertilization rate, cleavage rate, implantation rate, or TQE rate among the two groups [24]. Additionally, Pardinas et al conducted a retrospective analysis of 1,597 patients receiving a GnRH antagonist protocol and found that serum progesterone > 1.5 ng/mL on the day of trigger administration was not associated with the number of euploid embryos or TQE rate [25]. Few studies [15, 16] have assessed post-trigger progesterone, either the change in progesterone before and after trigger or absolute post-trigger progesterone level, and IVF cycle outcomes. Zhu et al stratified 2,978 IVF cycles into seven groups based on the ratio of change in progesterone from the time of trigger administration to 10–12 h after trigger administration and did not identify an association in progesterone change and oocyte or embryo quality [15]. Kummer et al performed a retrospective analysis of 508 autologous and oocyte donor cycles utilizing a GnRH antagonist protocol and a GnRH agonist to trigger oocyte maturation and found that peak E2 on the day of trigger, post-trigger LH and progesterone, and LH rise were positively correlated with the number of total and mature oocytes retrieved [16]. In keeping with prior studies, our analysis did not identify an association between post-trigger progesterone and oocyte maturation, blastulation, or fertilization rate. However, we did identify a correlation between low post-trigger progesterone levels and low oocyte recovery rate. Given that exogenous FSH promotes follicular development and induces LH receptor expression, follicles that are responsive to FSH should express more LH receptors, increasing their responsiveness to the trigger injection that initiates final oocyte maturation and ultimately facilitates oocyte recovery. This may, in part, explain the positive correlation between post-trigger progesterone levels and the number of retrieved oocytes [15, 16]. Conversely, poor FSH responsiveness may lead to inadequate LH receptor expression and decreased oocyte recovery rate, as demonstrated by our result. Taken together, the current literature suggests an association between progesterone level and cycle outcomes; however, further research is needed to elaborate on these associations.

This study has many strengths, including the minimization of inter-provider variability, given that monitoring, protocol adjustments, and procedures were all performed at a single site. Most importantly, we have examined previously understudied metrics of IVF cycle outcomes and, based on our data, suggest that post-trigger progesterone levels are a useful parameter associated with IVF cycle outcomes. This study is limited by its retrospective design, relatively small sample size, and lack of a priori power analysis. Furthermore, given the small sample size and high rate of oocyte cryopreservation in our patient population, we were unable to assess pregnancy and live birth outcomes, limiting our assessment of the broader clinical impact of our data. There is also the potential for unmeasured confounders that may have altered our study results, such as variations in gonadotropin dosing and follicular size distribution at time of trigger administration. Finally, additional analyses, including receiver operating characteristic (ROC) curves, were not performed because the clinical significance of our findings appeared to be limited. The significance of post-trigger progesterone levels should be studied in larger, prospective studies to better understand its value as a predictor of IVF cycle outcomes.

## Conclusions

This study sought to add to the existing literature by assessing changes in hormone levels on the day after trigger injection in protocol- and trigger-specific analyses. We found that low post-trigger progesterone levels are associated with poor oocyte recovery rates, but are not associated with oocyte maturity, fertilization or blastulation rate. Additionally, post-trigger estradiol levels are not associated with poor IVF outcomes. Based on our findings and the existing literature, post-trigger hormone levels should not be used to guide clinical decision-making. However, the small yet potentially clinically meaningful association between low post-trigger progesterone levels and reduced oocyte recovery warrants further investigation in larger, adequately powered studies.

## Figures and Tables

**Table 1:** Patient demographics and cycle outcomes

	All Cycles (n = 320)
**Age (years)**	
Range	25 - 48
Mean (SD)	37.6 (4.1)
**BMI**	
Range	17.54 - 70.52
Mean (SD)	27.7 (8.1)
**Race/Ethnicity, n (%)**	
African American	15 (4.7)
Asian	39 (12.2)
Caucasian	95 (29.7)
Hispanic	34 (10.6)
Unknown	137 (42.8)
**Number of Oocytes Retrieved**	
Range	1 - 47
Mean (SD)	11.0 (7.5)
**Oocyte Recovery Rate (proportion)**	
Range	0.23 - 2
Mean (SD)	0.9 (0.3)
**Number of Mature Oocytes**	
Range	0 - 30
Mean (SD)	7.2 (5.1)
**Oocyte Maturity Rate (proportion)**	
Range	0 - 1
Mean (SD)	0.7 (0.2)
**Number of Fertilized Oocytes**	
Range	0 - 25
Mean (SD)	6.2 (4.4)
Median [Q1, Q3]	6 [3, 8]
**Oocyte Fertilization Rate (proportion)**	
Range	0 - 1
Mean (SD)	0.9 (0.1)
**Number of Blastocysts**	
Range	0 - 16
Mean (SD)	2.9 (3.0)
**Blastulation Rate (proportion)**	
Range	0 - 1
Mean (SD)	0.5 (0.3)

**Table 2:** Frequency of poor IVF outcomes

	All Cycles (n = 320)
**Oocyte Recovery Rate <= 50%, n (%)**	
No	282 (88.1)
Yes	38 (11.9)
**Oocyte Maturity Rate <= 40%, n (%)**	
No	295 (92.2)
Yes	25 (7.8)
**Oocyte Fertilization Rate <= 75%, n (%)**	
No	262 (81.9)
Yes	55 (17.2)
Not Applicable	3 (0.9)
**Oocyte Blastulation Rate <= 20% [4+ Fertilized], n (%)**	n = 223
No	172 (77.1)
Yes	51 (22.9)

## Data Availability

The data supporting the findings of this study are available from the corresponding author upon reasonable request.

## Abbreviations

OS: ovarian stimulation
GnRH: gonadotropin-releasing hormone
IVF: *in vitro* fertilization
SART: the Society for Assisted Reproductive Technology
E2: estradiol
P4: progesterone
hCG: human chorionic gonadotropin
LH: luteinizing hormone
OHSS: ovarian hyperstimulation syndrome
FSH: follicle-stimulating hormone
MII: metaphase II
2PN: two pronucle
PGT: preimplantation genetic testing
SD: standard deviation
IQR: interquartile range
GEE: generalized estimating equations
BMI: body mass index
OR: odds ratio
CI: confidence interval
GQE: good quality embryo
TQE: top quality embryo
FET: frozen embryo transfer
